# A Case of Asthma Successfully Treated with Hypodermic Injections of Arsenic

**Published:** 1886-06

**Authors:** 


**A Case of Asthma Successfully Treated with Hypodermic Injections of Arsenic.**—A man, aged 30, suffering repeatedly with attacks of asthma, was treated for some time with various remedies, among them the hypodermic injections of morphia, but without relief. Finally Fowler's solution of arsenic in 10 minim doses, was injected under the skin with excellent success. After 12 injections the attacks ceased and the general health of the patient improved remarkably. A few months after apparant recovery, a recurrence took place, when two injections relieved him completely. These injections caused no unpleasant symptoms, and locally produced nothing worse than most any other injections, slight indurations, which disappeared after few hours. After the first two injections the patient complained of some pain in the leg of the injected side, which lasted but a very short time.—*Medical Central Zeitung.*

